# Effects of cooperative games on enjoyment in physical education—How to increase positive experiences in students?

**DOI:** 10.1371/journal.pone.0243608

**Published:** 2020-12-07

**Authors:** Eliane Stephanie Engels, Philipp Alexander Freund

**Affiliations:** Department of Psychology, Leuphana University of Lueneburg, Lueneburg, Germany; La Inmaculada Teacher Training Centre (University of Granada), SPAIN

## Abstract

Enjoyment is one of the most important factors for the maintenance of regular physical activity. The present study investigated if cooperative games in physical education classes (grades 6–9) can increase students’ enjoyment of physical activity. Data were collected in a quasi-experimental study employing a two-group design with repeated measures and randomization of classes to conditions. The total sample consisted of *N* = 285 students from regular schools in Germany aged 10 to 16 years (*M*_age_ = 12.67 years, *SD* = 1.10; 48.4% female). We found that cooperative games led to a higher perceived enjoyment in physical education classes (*F*(1) = 3.49, *p* = .063, *η*_*p*_^2^ = .012), increased the feeling of how strong students felt related to each other (*F*(1) = 4.38, *p* = .037, *η*_*p*_^2^ = .016), and facilitated feelings of perceived competence in physical education class (*F*(1) = 6.31, *p* = .013, *η*_*p*_^2^ = .022). In addition, social relatedness and perceived competence partly mediated the effect of cooperative games on enjoyment. The findings indicate that systematically designed cooperative games can help foster enjoyment in physical education classes.

## Introduction

Regular physical activity is crucial for a healthy life and positively affects overall mental and physical development [1]. However, many people face barriers participating in physical activity and exercising regularly [2]. Already in adolescence, a decline in physical activity has become apparent in recent decades. For example, only 12% of girls and 19.1% of boys aged 11 to 15 years in Germany achieve the recommended daily level of 60 minutes of physical activity for adolescents [3].

An important factor for the maintenance of regular physical activity is perceived enjoyment, e.g. [4,5]. Therefore, it seems desirable to encourage enjoyment of physical activity early on. Physical education classes constitute an important environment for the promotion of physical activity because basically everyone has to participate in them at school [6]. Empirical studies indicate that the more adolescent students enjoy participating in physical education classes, the more they engage in sports outside school as well [5,7].

A crucial prerequisite to optimally promote interest in and enjoyment of physical activity is gaining knowledge of which factors influence students’ perceptions of enjoyment in physical education. Previous research has provided evidence that social aspects and perceived competence are central factors to enjoying physical education [8–11]. Social aspects appear to be of the highest importance. They include being part of a group and in the company of others, creating close and warm relationships, and using communication [12]. These social aspects trigger enjoyment regardless of gender, age, or sports type [12]. The second most influential factor for enjoying sports is the perception of personal competence, which describes the feeling of being successful, achieving a goal, or making progress [12]. According to these findings, social aspects and perceived competence could be promoted by creating suitable interventions in physical education classes in order to enhance enjoyment. Unfortunately, physical education classes often focus on competitive sports types [13,14] and are often characterized by a rather strict input from teachers, a lack of choices for students, low perceived competence in many students, little social involvement caused by a lack of cooperation, and generally a bad climate for interaction [15]. Therefore, interventions for physical education appear to be a promising option to change this defective situation.

### Self-determination theory

Self-determination theory (SDT; Deci & Ryan [16]) is one of the most common theoretical frameworks to explain motivation and has also been investigated within the context of physical education [16]. SDT proposes three basic needs: the need for autonomy, the need for competence, and the need for social relatedness. If these psychological needs are satisfied, autonomous motivation develops according to SDT. Previous research has shown that the satisfaction of these three psychological needs is associated with experiencing more enjoyment in physical education [7,17,18]. Moreover, the experience of autonomy, competence, and social relatedness is a requirement for mental and physical well-being [19]. A review showed that intervention studies which integrate pedagogical and psychological knowledge are lacking in the context of physical education [20]. SDT offers a useful approach for the development of interventions to increase enjoyment in physical education by improving social interaction through creating a warm environment and facilitating the feeling of competence [20].

### Cooperative games

Cooperative learning approaches aim to promote positive social interaction by having students strive toward collective targets and work together as a team [13,21]. Following Huber [22], there are three essential elements of cooperative learning: a clearly defined group target with space for autonomous decisions, individual accountability, and positive interdependence [22]. Collective target achievement and problem-solving encompasses many interactions and requires effective and clear communication [23–25]. The better the perceived social interaction between students, the more they feel related to each other [26,27].

Previous research for cooperative learning approaches has found evidence for improvement of interpersonal skills and social competencies [28,29]. Johnson, Erwin, Kipp, and Beighle [30] showed that students enjoyed physical education significantly more if teachers stressed cooperative learning. According to Gilsdorf and Kistner [31], cooperative games focus on improving social interaction, collective target achievement, trust in each other, and feelings of success [31]. Competition is not relevant in these games [32,33]. Cooperative games require participants to think about consequences before they act [24]. Every student is an important part of the group’s success, which promotes the aspects of social relatedness and perceived competence. Therefore, cooperative games seem to be a promising way to encourage social relatedness and increase perceived competence in order to enhance enjoyment. Extant empirical findings support the favorable outcomes of using cooperative games in educational settings. Bay-Hinitz et al. [23] compared competitive and cooperative games and showed an increase in cooperative behavior stemming from cooperative games and an increase in aggressive behavior after engaging in competitive games for four to five year old children. The findings of Street et al. [24] showed that cooperative games increased pro-social behavior among primary school children.

In summary, there is a lack of research concerning interventions designed to enhance the enjoyment of physical education [20], and, thus far, there are no empirical studies investigating the effectiveness of cooperative games to foster enjoyment in adolescent students.

#### The present study

The purpose of the present study was to examine whether cooperative games can increase enjoyment in physical education classes for students aged 12 to 16 years. In this age group, the enjoyment of physical activity is lower than it is in childhood mainly due to changes in physical, cognitive, and social development [34]. Therefore, the enjoyment of physical activity and, at school, physical education classes, should be promoted especially in this age group [35]. We developed an intervention program based on previous findings [24,26–29] and theoretically grounded on SDT [16]. This intervention program consisted of short cooperative games (15 minutes each), which were systematically implemented in the beginning of physical education lessons. We assumed that participants in the cooperative-games condition would perceive more enjoyment in physical education classes than participants in the noncooperative-games condition (Hypothesis 1; cf. [20]). Furthermore, we expected participants in the cooperative-games condition to report higher feelings of social relatedness in physical education classes than participants in the noncooperative-games condition (Hypothesis 2; cf. [31]). The impact of cooperative games on the feeling of social relatedness should be moderated through perceived social interaction in physical education classes (e.g., [36]): The higher the perceived social interaction, the greater the effect regarding social relatedness (Hypothesis 3). Moreover, we expected that participants in the cooperative-games condition would report a higher perceived competence in the physical education class than participants in the noncooperative-games condition (Hypothesis 4; cf. [31]). Finally, for the cooperative-games condition, we assumed that the impact on enjoyment is mediated by the feeling of social relatedness (Hypothesis 5) and perceived competence (Hypothesis 6; cf. [9]). Thus, we investigated the effects of cooperative games on participants’ enjoyment, their social relatedness, and their perceived competence. Since findings of Engels and Freund [8] showed a weak influence of autonomy on enjoyment in physical education, we refrained from formulating specific assumptions concerning autonomy. However, for the sake of completeness regarding to theoretical assumptions in SDT, we investigated autonomy exploratively. As a further exploratory investigation, we examined the influence of potential gender differences.

This study was preregistered before data collection (https://osf.io/czgdx/).

## Methods

### Design

We conducted a quasi-experimental study to analyze the effects of cooperative games on enjoyment of physical education using a two-group design (intervention vs. control group) with repeated measures. We examined natural groups in which only randomization of classes to conditions (cooperative-games vs. noncooperative-games) was possible. Over a period of seven to fourteen weeks, physical education classes in the intervention condition engaged in 15-minute-cooperative games once per week, while classes in the control condition conducted regular physical education classes without any additional treatment. To prevent any potential disadvantages for the participants in the control condition, we recommended that these classes should receive the intervention in the following school year. We measured all variables in both groups at two time points: right before starting the intervention in week one (pretest) and after completion of all lessons in week 14 (posttest).

#### Intervention

The development of the intervention program was based on previous research regarding cooperative learning approaches [24,28,29], components of existing cooperative games [31] and led by SDT [16]. We selected cooperative games that aim to improve the most important factors for increasing the enjoyment of physical education, such as the feelings of social relatedness and perceived competence in physical education. To reach these goals, we followed the structural approach of cooperative learning and chose games that foster the two main elements: positive interdependence to group members and individual accountability [37], for example, *Blinded Soccer and Pyramid Construction*. These games focus especially on communication, cooperative coordination, collective problem solving, mutual dependence tolerance, physical contact, trust, collective strategy building, and planning.

To evaluate the first version of the intervention program, we conducted a prestudy with *N* = 58 students (*M*_*age*_ = 11.0 years, *SD* = 0.53; female: 42.9%) at the fifth class level. We tested 16 cooperative games regarding their practicability for teachers and their effects on enjoyment, perceived competence, social relatedness, and social interaction over 16 weeks/lessons. The key findings of this prestudy showed that cooperative games are viable tools for increasing the enjoyment of physical education in students and that participants in the intervention group reported a higher feeling of social relatedness and a higher perceived competence in physical education classes.

To develop the final intervention program, we selected the games that were judged to be most easily implemented by teachers and most enjoyable for students. We asked teachers which games they deemed fit for the given time limit of approximately fifteen minutes each and for which the standardized instructions were clear and understandable for all students. The teachers named two games (*Steeplechase* and *Trust Run*) in which they encountered some problems. Thus, these games were removed. The final intervention program consisted of 14 cooperative game units. Due to organizational requirements from several schools, we were obliged to reduce the program to conduct a shorter version. Therefore, we divided the program into two similar versions with seven games each. In the end we used two versions: a short version with 7 games and a long version with 14 games. An overview of the complete intervention program is given in S1 Table.

All games can be conducted with minimal equipment and standardized instructions. Teachers were able to implement the games without any external help. Every game requires less than fifteen minutes so that they are compatible with other teaching contents of the lesson. The intervention was conducted in regular physical education lessons (in this case, 90 minutes sessions). The games were systematically implemented at the beginning of each lesson and were carried out weekly.

#### Procedure

The research program was reviewed and approved beforehand by an ad hoc institutional review board (Institute of Psychology, Leuphana University of Lueneburg, Germany). The study design, the means of data collection, and all methods of data processing were registered and approved by this board. Students and parents were comprehensively informed in advance about the objectives and the procedure of the study.

Experiences in physical education were assessed with a paper-pencil questionnaire. Permission to hand out the questionnaire to students was obtained from the school principals and written parental consent was secured by signature for all participating students. Students were informed that participation in this survey was voluntary and anonymous. Students completed the questionnaires during regular lessons (approximately 5 to 10 minutes). Participation in the intervention program (i.e., cooperative games) as part of physical education classes was obligatory. The recruitment of teachers was accomplished via e-mail. We contacted 27 physical education teachers and asked them to participate in this study with at least two parallel classes. Nine teachers agreed to participate. An information letter described our research project in general. We aimed for all teachers to participate with at least two classes (intervention and control) of the same class level to control the influence of individual teaching style. In the end, we could not realize this goal for all teachers due to organizational restrictions and unexpected cancellations by schools. Six teachers taught in both conditions and three only in one conditions (two in cooperative-games and one in noncooperative-games). Data from two complete classes that took part in less than half of the intervention lessons were therefore excluded from the analyses.

### Sample

Sample size was determined a priori using G*Power 3.*1*.*9*.*2* [38]. Based on previous research on cooperative learning approaches, e.g. [28], we assumed a moderate effect size along with a minimum of 80% statistical power. The following parameters were preregistered (see https://osf.io/czgdx/) and entered in G*Power: *f* = 0.30, α = 0.05, 1-β = .80. The minimum total sample size was estimated as *N* = 190 participants. We were able to recruit a total sample size of *N* = 285 participants from regular schools (comprehensive schools, secondary schools and grammar schools) in the federal state of Lower Saxony (Germany). Participants were students from grades six to nine in the age range of 10 to 16 years (*M*_*age*_ = 12.67 years, *SD* = 1.10; 48.4% female). For the total sample, the intervention group consisted of *n*_1_ = 155 students (*M*_*age*_ = 12.77 years, *SD* = 1.21; 44.5% female) from eight classes (all taught by different teachers), and the control group included *n*_2_ = 130 students (*M*_*age*_ = 12.55 years, *SD* = 0.94; 53.71% female) from seven classes. The total sample consisted of two independent subsamples: The first sample included *N* = 143 students (*M*_*age*_ = 12.5 years, *SD* = 1.50; female: 43.4%) of class levels from 7 to 10. These students participated either in the 7-week intervention program or in the corresponding control group. Participants of the second sample were *N* = 142 students (*M*_*age*_ = 12.85 years, *SD* = 1.34; female 53.5%), these either took part in the 14-week intervention program or in the corresponding control group. The combination of these two samples led to a higher sample size than estimated. For more information about the sample distribution, see Table 1.

**Table 1:** Distribution of sample regarding gender, age and class level.

Program	Group		Gender (%)		Age (years)		Class level (%)			
		*N*	female	male	*M*	*SD*	6	7	8	9
Complete	IG	155	44.5	55.5	12.77	1.21	27.7	47.1	10.3	14.8
	CG	130	53.1	46.9	12.55	0.94	28.5	45.4	26.2	0
	Total	285	48.4	51.6	12.67	1.10	28.1	46.3	17.5	8.1
7 weeks	IG	80	41.3	58.8	12.58	0.79	50.0	50.0	00.0	00.0
	CG	63	46.0	54.0	12.40	0.58	33.3	66.6	00.0	00.0
	Total	143	43.4	56.6	12.50	0.71	28.7	71.4	00.0	00.0
14 weeks	IG	75	48.0	52.0	12.99	1.52	30.7	17.3	21.3	30.7
	CG	67	59.7	40.3	12.70	1.17	23.9	25.4	50.7	00.0
	Total	142	53.5	46.5	12.85	1.34	27.5	21.1	35.2	16.2

*Note*. IG = Intervention group; CG = Control group.

### Measures

Enjoyment of physical education was measured by three facets (pleasure, flow experience, recovery) with the *Questionnaire for the Assessment of Enjoyment in Physical Education* (*QUAEPE;* Engels & Freund [39,40]). Pleasure is related to the affective experience in physical education (e.g., “Physical education is fun“). Flow experience describes the optimal perceived process during physical education lesson (e.g., “In physical education, I feel optimally strained“). Recovery defines how students feel balanced, relaxed, and vitalized by physical education (e.g., “Physical education gives me energy for other things“). All scales consisted of three items each, and responses were assessed on a 4-point-rating scale (0 = *never*, 1 = *sometimes*, 2 = *often*, 3 = *always*). For more information about the development and validation of scales and items to measure students’ enjoyment in physical education, see Engels and Freund [39] as well as Lohbeck et al. [40].

Social relatedness, social interaction, perceived competence, and autonomy were measured with scales developed and presented by Engels and Freund [8]. Social relatedness describes how much the students feel integrated and involved in their class during physical education (e.g., “In physical education, I feel connected to my classmates“). Social interaction refers to cooperation in class during physical education (e.g., “In physical education, we treat each other fairly“). Perceived competence is related to the self-experienced competence in physical education (e.g., “I am good at physical education“). Autonomy describes the extent of possibilities for co-determination and co-designing physical education class (e.g., “In physical education class we are allowed to participate in designing lessons“). The four variables (social relatedness, social interaction, perceived competence, and autonomy) were measured by three items each and rated on a 4-point rating scale ranging from *0 (never) to 3 (always)*. All items used in the present study are available in German (original) and English language in S2 Table. For additional information on quality criteria and item validation, see Engels and Freund [8]. Additionally, as fidelity check after completion of the intervention program, physical education teachers were asked whether they had carried out the complete program in the predefined way, using the standardized instructions for the games.

### Data analyses

Scale means and standard deviations are reported for the total sample and separately for the intervention and control groups for both times of measurement. We analyzed whether any of the dependent variables differed significantly between the intervention group and the control group at the first time of measurement before the intervention was initiated with independent *t*-tests. To determine the effects of the intervention on students’ enjoyment (Hypothesis 1), social relatedness (Hypothesis 2), perceived competence (Hypothesis 4), and exploratory for autonomy we conducted an analysis of variance (ANOVA) with repeated measures. Here, we included the following covariates: type of intervention program, student gender, class level, and type of school. As post-hoc corrections of multiple comparisons, we used Bonferroni correction to avoid the accumulation of the alpha error. To test Hypothesis 3 regarding the moderating impact of social interaction in class on the effect of social relatedness, we conducted a moderation analysis using *Process macro 3*.*1* (Hayes [41]; Model 1). With two simple mediation analyses, we tested whether and to what extent social relatedness (Hypothesis 5) and perceived competence (Hypothesis 6) account for the effect on enjoyment. We ran bootstrapping procedures with 5,000 iterations (*Process macro 3*.*1*; Hayes [41]; Model 4).

## Results

### Descriptive statistics and primary analyses

Internal consistencies (Cronbach’s alpha) ranged from acceptable to good for all scales. The complete enjoyment scale showed the same good internal consistencies for both times of measure (α = .89). For the subscales of the questionnaire, we obtained the following estimates of internal consistency: pleasure: α_*t1*_ = .83, α_*t2*_ = .83; flow: α_*t1*_ = .68, α_*t2*_ = .60; recovery: α_*t1*_ = .79, α_*t2*_ = .82; social relatedness: α_*t1*_ = .76, α_*t2*_ = .70; social interaction: α_*t1*_ = .76, α_*t2*_ = .73; perceived competence: α_*t1*_ = .78, α_*t2*_ = .78); α_*t2*_ = .73; autonomy: α_*t1*_ = .77, α_*t2*_ = .84).

Overall, we found the highest scale means for pleasure (*M* = 2.18; *SD* = 0.69) for the intervention group at *t*_*2*_ and the lowest value for social relatedness (*M* = 1.38; *SD* = 0.67) for the control group at *t*_*2*_ (see Table 2). Independent *t*-tests showed that there were already significant (*p* < .05) differences between the intervention and control groups at the first time of measurement (*t*_*1*_) regarding all dependent variables besides recovery (*t*(283) = 1.17, *p* = .241) and perceived competence (*t*(283) = 0.47, *p* = .637). For that reason, we controlled for the dependent variables at *t*_*1*_ in all further analyses.

**Table 2:** Scale means, standard deviation for intervention and control group for both times of measurement.

Variable	Group	*t*_*1*_		*t*_*2*_	
		*M*	*SD*	*M*	*SD*
Enjoyment of PE	IG	1.88	0.68	2.03	0.63
	CG	1.71	0.65	1.74	0.62
Pleasure	IG	2.01	0.76	2.18	0.69
	CG	1.79	0.72	1.83	0.71
Flow	IG	1.89	0.67	2.03	0.61
	CG	1.70	0.71	1.71	0.60
Recovery	IG	1.75	0.88	1.87	0.82
	CG	1.63	0.80	1.67	0.81
Social relatedness	IG	1.56	0.66	1.70	0.70
	CG	1.40	0.68	1.38	0.67
Social interaction	IG	1.84	0.63	1.97	0.53
	CG	1.56	0.70	1.55	0.63
Perceived competence	IG	1.67	0.71	1.80	0.72
	CG	1.63	0.69	1.63	0.64
Autonomy	IG	1.38	0.68	1.60	0.67
	CG	1.15	0.60	1.46	0.70

*Note*. *PE = physical education; t*_*1*_ = first time of measure; *t*_*2*_ = second time of measure; IG = intervention group, CG = control group.

### Hypotheses testing

All interaction and main effects for Hypothesis 1, Hypothesis 2, and Hypothesis 4 are reported in Table 3. For Hypothesis 1, regarding the effects of cooperative games on enjoyment, we found only a small, marginally significant effect for the interaction of group and time. Enjoyment increased more strongly in the intervention group than in the control group. The findings for the subscale pleasure also showed a small and marginally significant effect for the interaction between group and time. This indicated that the intervention had a specific effect on students’ perceived pleasure in physical education. Regarding flow and recovery, we found no effects for the interaction between group and time on the experience of flow or the feeling of recovery. These results indicate that the intervention had no specific impact on students’ feelings of flow and recovery in physical education. The observed main effects of group regarding enjoyment and its facets show that participants in the cooperative-games condition reported more enjoyment in physical education class than participants in the noncooperative-games condition, independent of time of measurement.

**Table 3:** Effects of interaction testing Hypothesis 1, Hypothesis 2, Hypothesis 4, and exploratory for autonomy as well as main and time effects.

Dependent variable	Effects	*F*	*df*	*p*	*η*_*p*_^*2*^
Enjoyment of PE	Group × Time	3.49	1	.063	.012
	Group	18.20	1	.001	.061
	Time	0.03	1	.855	.000
Pleasure	Group × Time	3.29	1	.071	.012
	Group	20.54	1	.000	.069
	Time	0.42	1	.518	.002
Flow	Group × Time	2.50	1	.115	.009
	Group	23.14	1	.001	.077
	Time	0.39	1	.531	.001
Recovery	Group × Time	0.89	1	.345	.003
	Group	5.59	1	.019	.020
	Time	0.14	1	.707	.001
Social relatedness	Group × Time	4.38	1	.037	.016
	Group	15.18	1	.001	.052
	Time	0.51	1	.474	.002
Perceived competence	Group × Time	6.31	1	.013	.022
	Group	2.30	1	.130	.008
	Time	1.42	1	.234	.005
Autonomy	Group × Time	1.11	1	.293	.004
	Group	13.24	1	.001	.046
	Time	4.92	1	.027	.017

*Note*. Group × Time = interaction between group and time; Group = intervention group vs. control group; time = first time of measure vs. second time of measure.

Considering the analyzed covariates, we found a large significant effect for class level (*F*(1) = 53.47, *p* < .001, *η*_*p*_^*2*^ = .161) and a small effect for type of intervention program (*F*(1) = 17.26, *p* < .001, *η*_*p*_^*2*^ = .058) on enjoyment. As an exploratory investigation, we also analyzed potential gender differences regarding the effects of cooperative games on enjoyment with the three subscales, but we did not find any significant interaction effects for group or gender.

As assumed in Hypothesis 2, students who took part in the cooperative-games condition reported a higher feeling of social relatedness in the physical education class than participants in the noncooperative-games condition at the second time of measurement. The ANOVA with repeated measures revealed a small and significant interaction between time and group: Social relatedness increased more strongly in the intervention group than in the control group. Additionally, there was a small and significant main effect for group, but not for time. However, we also observed small, significant main effects for the covariates class level (*F*(1) = 18.44, *p* < .001, *η*_*p*_^*2*^ = .062) and type of intervention program (*F*(1) = 4.18, *p* < .05, *η*_*p*_^*2*^ = .015) on students’ feelings of social relatedness.

To test Hypothesis 3, we conducted a moderation analysis to determine whether the impact of cooperative games on the feeling of social relatedness was moderated through the perceived social interaction in physical education class. However, the data did not support this hypothesis (*β* = .133, SE = .115, *p* = .246).

As expected in Hypothesis 4, we found that participants in the cooperative-games condition reported a higher perceived competence in physical education class than participants in the noncooperative-games condition. We found a small and significant interaction effect between group and time. It is therefore concluded that the intervention enhanced students’ perceived competence to a small degree.

Concerning Hypothesis 5, the results for the mediation analysis regarding social relatedness showed a significant total effect from cooperative games on enjoyment (*b* = .201, *SE =* 0.062, *p* = .001, BC CI_95%_ [+0.078; +0.234]). There was a significant direct effect from the intervention on enjoyment (*b* = .129, *SE =* 0.056, *p* = .033, BC CI_95%_ [+0.011; +0.247]) as well as from the intervention on social relatedness (*b* = .211, *SE =* 0.063, *p* < .001, BC CI_95%_ [+0.884; +0.3346]). The indirect effect of social relatedness on enjoyment was also significant (*b* = .072, *SE =* 0.025, BC CI_95%_ [+0.272; +0.125]). This means that 35.96% of the total effect from cooperative games on enjoyment can be explained by the indirect effect.

Regarding Hypothesis 6, we found a total effect from cooperative games on enjoyment (*b* = .267, *SE =* 0.065, *p* = .001, BC CI_95%_ [+0.139; +0.395]). We observed direct effects from the intervention on enjoyment (*b* = .203, *SE =* 0.062, *p* = .001, BC CI_95%_ [+0.082; +0.325]) and on perceived competence (*b* = .133, *SE =* 0.049, *p* = .007, BC CI_95%_ [+0.373; +0.229]). Additionally, we found a significant indirect effect (*b* = .064, *SE =* 0.024, BC CI_95%_ [+0.018; +0.113]). The indirect effect accounted for 23.82% of the total effect. Therefore, the effect of the intervention on enjoyment was partly mediated by social relatedness and perceived competence.

As another exploratory examination, we tested if the cooperative games showed an impact on autonomy using an ANOVA with repeated measures. Findings did not show an interaction effect between group and time. There were only significant main effects for group and time, meaning that there were already differences between the intervention and the control group from the beginning and autonomy increased over time in both groups. Therefore, it can be concluded that the intervention itself had no specific impact on the experience of autonomy in physical education class in students.

## Discussion

The present study aimed to evaluate an efficient intervention program utilizing cooperative games, which was systematically implemented at the beginning of physical education classes. The primary purpose of the intervention was to increase students’ enjoyment of physical education. We investigated cooperative games regarding their effects on enjoyment, social relatedness, perceived competence and, in an exploratory fashion, autonomy. The key results of the present study indicate a small direct effect of cooperative games on enjoyment and small indirect effects on enjoyment mediated by social relatedness and perceived competence.

We found some support for Hypothesis 1, in which we assumed that cooperative games would lead to a higher perceived enjoyment of physical education classes. Our results displayed a small effect of cooperative games on enjoyment, meaning that cooperative games led to a higher perception of enjoyment in students to some extent. These results are in line with the findings of Johnson et al. in the U.S., which showed that cooperative learning was associated with more enjoyment in physical education [30]. For the different facets of enjoyment, we could see that students who participated in the intervention program reported more pleasure in physical education, but the intervention had no specific impact on students’ feelings of flow and recovery in physical education. Thus, cooperative games can help to experience more fun and pleasure in physical education class, but these games do not contribute that students feel more recovered or relaxed. Various reasons could be responsible for the small effects. For example, the dose of the intervention might have been too low, since the cooperative games were used for only 15 minutes per lesson. Another explanation could be that the intervention is not equally effective for all students, but has an effect in specific target groups. Cooperative games may work better with students who usually perceive less enjoyment in physical education, or with students who are less competitively oriented. Therefore, preferences for cooperative and for competitive games may also be considered.

In Hypothesis 2, we expected that cooperative games would contribute to an increase in feelings of social relatedness in physical education class. The significant interaction between groups and time indicates a specific impact of cooperative games on the feeling of how strongly students feel related to each other in physical education. This finding is in line with previous research of other cooperative learning approaches, such as the improvement of cooperating skills [28] and social competencies [29].

In Hypothesis 3, we assumed that the effect of cooperative games on social relatedness would be moderated by social interaction. However, there was no interaction effect between groups and social interaction observed for social relatedness. Better social interaction between students in physical education class could not foster a stronger impact of cooperative games on social relatedness in students. This finding could be explained, for instance, by the fact that social interaction can also be seen as the outcome of a high social relatedness instead of a moderator variable. However, we could see that social relatedness is strongly related to social interaction. It is likely that there is an influence in both directions, i.e., a reciprocal relationship. The better the social interaction between students, the more they feel related to each other and as a consequence, they interact in a better way, eventually resulting in a positive circle. Moreover, a more negative climate in class at the beginning could lead to a stronger effect from cooperative games on social interaction because students can benefit more from the intervention. However, this needs to be investigated in future research and social relatedness should be tested as a mediator in the relationship between cooperative games and social interaction.

We found evidence for Hypothesis 4 that cooperative games lead to a higher perceived competence in students. Thus, students who participated in the cooperative games reported more perceived competence compared to students who did not. This finding could be explained by the idea that cooperative games especially help less skilled students to feel more successful and competent. This shows that cooperative games seem to be an important tool in physical education to integrate students who usually “get lost” in competitive games.

The exploratory analysis regarding the effect from cooperatives games on autonomy revealed no significant finding. This shows that the cooperative games appear to not make students feel more autonomous or self-determined. Contrary to the idea that cooperative approaches provide space for autonomous decisions [22], the cooperative games that we used in this study could not produce this effect. Potential explanations could lie in the way autonomy is measured in the present study or in the specific kind of cooperative games employed here.

As expected in Hypothesis 5, enjoyment was partly mediated by social relatedness. There was a small direct effect from the intervention on enjoyment as well as an indirect effect through social relatedness. Likewise, we found that the effect of cooperative games was partly mediated by perceived competence, as assumed in Hypothesis 6. A small significant indirect effect was observed in addition to the direct effect of the intervention on enjoyment. Consequently, it can be said that on the one hand, students benefit directly from cooperative games in that they perceive more fun and feel more skilled and more connected to each other in physical education, and on the other hand, cooperative games increase enjoyment via the positive experience of competence and affiliation (see, e.g., [8]). But these findings can only explain to some extent which factors contribute to a higher perception of enjoyment. Therefore multiple mediation analyses could be useful.

As an exploratory investigation, we also analyzed potential gender differences regarding the effects of cooperative games on enjoyment, including its three facets. However, we did not observe any significant interaction effects of groups and gender, meaning there were no differentiated effects regarding the efficacy of the intervention program between boys and girls.

### Limitations and directions of future research

The interpretation of the results of our study is restricted by some methodological limitations. To enhance power, we decided to combine the two independent samples from the short and the long version of the intervention program. Considering the analyzed covariates, we found significant main effects of class level and of intervention type on enjoyment. It can therefore be deduced that the class level and the type of intervention program itself had an impact on students’ perceived enjoyment. Further analysis (ANOVA with repeated measures) showed that there are differences between both programs regarding their effects on the dependent variables (S3 Table). Interestingly, the short program worked better than the long program. These differences might be explained by the length of the program itself: The effect of cooperative games might wear off after some time. Specifically, their novelty effect may already decrease after a few weeks, which is why the 7-week-program could have a better effect compared to the 14-week-program. It seems possible that the new experience and the more diversified physical education class also contributed to the increase of enjoyment. It was not possible to eliminate this influence because there was no control group included in our study that received another type of intervention. Another reason for these differences may be due to the sample used in each of the two types of program. The sample that participated in the shorter form of intervention consisted of sixth and seventh grades, while the long form of intervention also included eighth and ninth grades (see Table 1). Therefore, the specific games used in this study may work better with younger students than with older students. Previous research showed that younger students experience more enjoyment than older students anyway [39]. Hence, future studies should look more detailed at the reasons for these differences and may investigate separately the length of the intervention program and different class levels.

Since students are nested in classes, forthcoming studies should analyze the data within a multiple-group-multilevel design. Therefore, recruiting a larger sample with significantly more classes than in the present study seems a worthwhile pursuit. Data from a more complex sample might also help to understand the underlying mechanisms better, e.g., if the intervention has different effects on students depending on their class level.

Moreover, because we examined school classes, no randomization of individual participants to conditions was possible. As we tried to assign classes randomly to both conditions, the teachers ultimately decided by themselves in which class they conducted the intervention, given the requirement that they participated with at least two classes. It was also not possible to realize a follow-up measure to assess the long-term effect of the intervention because of organizational difficulties and time constraints in schools. While we had planned for each teacher to take part with at least two classes (one in the intervention group and one in the control group), this was not feasible under the circumstances met due to cancellations at short notice by the schools. Consequently, we could not control for the teaching style or the influence of the teacher and if the lesson content in intervention and control group were exactly the same apart from the cooperative games. In future studies a better control for teaching style is needed. One option would be to measure the individual teaching style with an additional instrument. Moreover, it seems necessary to control for the teaching contents in the intervention and control group. In addition, it would also be useful to measure the intensity of the movement and the subjective load, as this could also have an influence on the experienced enjoyment.

## Conclusion

In conclusion, it can be stated that cooperative games increase the perception of enjoyment of physical education in students to some extent. Participants in the intervention reported a higher feeling of social relatedness and a higher perceived competence in physical education class. Our findings thus indicate that systematically implemented cooperative games can provide support for a positive experience of physical education class in specific settings.

## Supporting information

S1 Table Overview of the complete intervention program. (DOCX)

S2 Table Items of the Questionnaire for the Assessment of Enjoyment in Physical Education (QUAEPE). (DOC)

S3 Table Separate analysis of effects for the 7-week program and the 14-week program. (DOCX)
