# Supplementary material for: Effects of cooperative games on enjoyment in physical education—How to increase positive experiences in students?
Source: PLoS One. 2020 Dec 7;15(12):e0243608. doi: 10.1371/journal.pone.0243608 (PMC7721155; doi:10.1371/journal.pone.0243608)
Supplement: S1 Table — (DOCX) [file pone.0243608.s001.docx]

S1 Table*.* Overview of the complete intervention program.

| **Name** | **Learning Objectives** | **Lesson Content** |
| --- | --- | --- |
| 1. The Magical Hoops | Cooperative Coordination | Forming a circle, holding hands. Climbing through plastic hoops without letting go. |
| 1. Mississippi | Cooperation, Communication | Groups will need to cross the gym without touching the floor, using two mats. |
| 1. Chimney Sweep | Cooperation, Communication | Groups form a circle, putting their arms around each other’s shoulders. Goal is to lift a ball up from the floor and out of the circle. |
| 1. Blanket Turnaround | Cooperation, Tolerance, Communication, Collective Problem Solving | Groups sit on a blanket. Goal is to turn over the blanket without touching the floor. |
| 1. Change of Places | Communication, Collective Strategy Building, Mutual Dependence, Physical Contact | Groups stand on a gymnastic bench. Goal is to swap positions based on age, shoe size etc. without touching the floor. |
| 1. Space in the Smallest Hut | Communication, Collective Planning and Cooperation | Groups form a small circle out of a rope, standing inside of it. Teacher specifies how many feet, hands etc. can touch the floor. |
| 1. Human Knot | Cooperation, Communication, Physical Contact | Form a circle. Everyone blindly grabs hands of two other players. Goal is to untangle the human knot. |
| 1. 7 People with 4 Feet | Cooperation, Communication | Groups of 7 will need to cross a distance of 10 meters, only having 4 body parts on the ground. |
| 1. Siamese Soccer | Cooperation, Communication | Two teams play soccer against each other. Players will be paired up, tying one foot to the foot of another player. |
| 1. Transportation on a Conveyor Belt | Trust, Cooperation | Groups (boys and girls separated) lie down on their backs. Students lying next to each other need to point their legs to different directions. Now they carry one student to the other side. |
| 1. Save me if you can! | Physical Contact, Cooperativeness | Students will be allocated to group 1, 2 or 3. They dance until the music stops. Teacher calls out a number. Those students will sink to the floor. Other students will try to save them. When music continues, “fallen students” can be saved by hugging them. |
| 1. Free Fall | Trust, Cooperation | A student stands on a box, letting him or herself drop back. Other students catch him or her, holding a blanket between them. |
| 1. Blinded Soccer | Trust, Cooperation, Communication | Teams consist of pairs. One partner is blind-folded, the other guides the “blind player” through the soccer game by instructing him or her how to pass the ball. |
| 1. Pyramid Construction | Trust, Cooperation, Communication | Goal is to build a pyramid out of 7 people. |

*Note.* For the short version of the program (7 weeks) we divided the program into two equal halves: 1. version included the games: *The magical Hoops, Chimney Sweep, Change of Places, Human Knot, Siamese Soccer, Save me if you can, Blinded Soccer*; 2. version included the games: *Mississippi, Blanket Turnaround, Space in the Smallest Hut, 7 People with 4 Feet, Transportation on a Conveyor Belt, Free Fall, Pyramid Construction*.
