# Supplementary material for: Effects of cooperative games on enjoyment in physical education—How to increase positive experiences in students?
Source: PLoS One. 2020 Dec 7;15(12):e0243608. doi: 10.1371/journal.pone.0243608 (PMC7721155; doi:10.1371/journal.pone.0243608)
Supplement: S2 Table — (DOC) [file pone.0243608.s002.doc]

**S2 Table. Items of the Questionnaire for the Assessment of Enjoyment in Physical Education (QUAEPE)**

| Item | Content | | Original (German) | |
| --- | --- | --- | --- | --- |
| **Pleasure** | |  | |  |
| 1. | Physical education is fun. | | Sportunterricht macht mir Spaß. | |
| 2. | Physical education makes me happy. | | Sportunterricht macht mich fröhlich. | |
| 3. | Physical education is good for me. | | Sportunterricht tut mir gut. | |
| **Flow** |  | |  | |
| 4. | In physical education, there are activities that excite me. | | Im Sportunterricht gibt es Aktivitäten, die mich begeistern. | |
| 5. | In physical education, time flies. | | Im Sportunterricht vergeht die Zeit wie am Flug. | |
| 6. | In physical education, I feel optimally strained. | | Im Sportunterricht fühle ich mich optimal beansprucht. | |
| **Recovery** | |  | |  |
| 7. | In physical education, I can recover from other subjects. | | Im Sportunterricht kann ich mich von anderen Fächern erholen. | |
| 8. | Physical education helps me to clear my mind. | | Sportunterricht hilft mir, den Kopf frei zu kriegen. | |
| 9. | Physical education gives me energy for other things. | | Sportunterricht gibt mir Energie für andere Dinge. | |
| **Social relatedness** | |  | |  |
| 10. | In physical education, I feel connected to my classmates. | | Im Sportunterricht erlebe ich ein Gemeinschaftsgefühl. | |
| 11. | Physical education helps me to clear my mind. | | Im Sportunterricht fühle ich mich mit meinen Mitschülern verbunden. | |
| 12. | In physical education, I have a sense of belonging. | | Im Sportunterricht habe ich das Gefühl von Zusammengehörigkeit. | |
| **Social interaction** | |  | |  |
| 13. | In physical education, we can rely on each other. | | Wir können uns im Sportunterricht aufeinander verlassen. | |
| 14. | In physical education, we treat each other fairly. | | Wir gehen im Sportunterricht fair miteinander um. | |
| 15. | In physical education, we look out for each other. | | Wir nehmen im Sportunterricht Rücksicht aufeinander. | |
| **Perceived competence** | |  | |  |
| 16. | I am good in physical education. | | Ich bin im Sportunterricht gut. | |
| 17. | In physical education, new exercises are easy for me. | | Mir fallen neue Übungen im Sportunterricht leicht. | |
| 18. | In physical education, I am doing better than many of my classmates. | | Ich bin im Sportunterricht besser als viele meiner Mitschüler. | |
| **Autonomy** | |  | |  |
| 19. | In physical education we are free to have a say in what we do. | | Im Sportunterricht dürfen wir mitbestimmen, was wir machen. | |
| 20. | In physical education class we are allowed to participate in designing lessons. | | Wir dürfen die Sportstunde mitgestalten. | |
| 21. | Our teacher responds to our wishes in sports lessons. | | Unser Lehrer geht im Sportunterricht auf unsere Wünsche ein. | |

*Note.* Items are answered using a 4-point rating scale ranging from 0 (*never*) to 1 (*sometimes*) to 2 (*often*) to 3 (*always*). For more information regarding development and validation of items and scales, see: Engels & Freund (2018); Engels & Freund (2019); Lohbeck, Engels, & Freund (2018).
