# Supplementary material for: Effects of cooperative games on enjoyment in physical education—How to increase positive experiences in students?
Source: PLoS One. 2020 Dec 7;15(12):e0243608. doi: 10.1371/journal.pone.0243608 (PMC7721155; doi:10.1371/journal.pone.0243608)
Supplement: S3 Table — (DOCX) [file pone.0243608.s003.docx]

**S3 Table. Separate analysis of effects for the 7-week program and the 14-week program.**

|  |  | **7-week program** | | | | **14-week program** | | | |
| --- | --- | --- | --- | --- | --- | --- | --- | --- | --- |
| **Dependent variable** | **Effects** | ***F*** | ***df*** | ***p*** | ***η_p_^2^*** | ***F*** | ***df*** | ***p*** | ***η_p_^2^*** |
| Enjoyment of PE | Group × Time | 9.10 | 1 | .003 | .062 | 0.01 | 1 | .906 | .000 |
|  | Group | 3.24 | 1 | .074 | .023 | 17.78 | 1 | .001 | .115 |
|  | Time | 0.10 | 1 | .750 | .001 | 0.01 | 1 | .919 | .000 |
|  | Group × Time | 5.13 | 1 | .025 | .036 | 0.21 | 1 | .650 | .002 |
| Pleasure | Group | 5.79 | 1 | .017 | .040 | 16.55 | 1 | .001 | .108 |
|  | Time | 1.49 | 1 | .239 | .010 | 0.12 | 1 | .911 | .000 |
|  | Group × Time | 7.08 | 1 | .009 | .049 | 0.06 | 1 | .804 | .000 |
| Flow | Group | 3.92 | 1 | .050 | .028 | 25.11 | 1 | .001 | .115 |
|  | Time | 0.37 | 1 | .544 | .003 | 1.41 | 1 | .708 | .001 |
|  | Group × Time | 3.84 | 1 | .052 | .027 | 0.33 | 1 | .568 | .002 |
| Recovery | Group | 0.19 | 1 | .663 | .001 | 8.11 | 1 | .005 | .056 |
|  | Time | 0.57 | 1 | .811 | .000 | 0.04 | 1 | .836 | .000 |
| Social relatedness | Group × Time | 9.27 | 1 | .003 | .063 | 0.10 | 1 | .756 | .001 |
|  | Group | 1.24 | 1 | .267 | .009 | 22.07 | 1 | .001 | .139 |
|  | Time | 0.00 | 1 | .992 | .000 | 0.74 | 1 | .392 | .005 |
| Perceived competence | Group × Time | 2.39 | 1 | .124 | .017 | 3.82 | 1 | .053 | .027 |
|  | Group | 1.97 | 1 | .163 | .014 | 0.38 | 1 | .540 | .003 |
|  | Time | 0.16 | 1 | .691 | .001 | 2.38 | 1 | .125 | .017 |
|  | Group × Time | 0.88 | 1 | .351 | .006 | 4.58 | 1 | .034 | .032 |
| Autonomy | Group | 0.57 | 1 | .453 | .004 | 19.86 | 1 | .001 | .127 |
|  | Time | 0.45 | 1 | .506 | .003 | 0.65 | 1 | .420 | .005 |

*Note.* Group × Time = interaction between Group and Time; Group = intervention vs. control group; time = first time of measure vs. second time of measure; *PE = physical education*.
